# Complications of Advanced Kadish Stage Esthesioneuroblastoma: Single Institution Experience and Literature Review

**DOI:** 10.7759/cureus.1245

**Published:** 2017-05-12

**Authors:** Sheri K Palejwala, Saurabh Sharma, Christopher H Le, Eugene Chang, Michael Lemole

**Affiliations:** 1 Neurosurgery, University of Arizona; 2 Otolaryngology, Banner University Medical Center - Tucson, Main Campus; 3 University of Arizona

**Keywords:** esthesioneuroblastoma, olfactory neuroblastoma, skull base, sinonasal, complications, kadish stage, reconstruction

## Abstract

**Introduction:**

In esthesioneuroblastoma, greater disease extent and Kadish staging correlate with greater recurrence, complications, and mortality. These advanced stage malignancies require extensive resections and aggressive adjuvant therapy. This increases the risk of complications such as cerebrospinal fluid leak, neurologic deficits, and osteomyelitis. We present our case series and then analyze the literature to ascertain whether advanced stage tumors corresponds to greater rates of complications.

**Methods:**

A retrospective review of consecutive patients with histologically-proven esthesioneuroblastoma who were aggressively managed at our institution was performed. This was followed by an extensive literature search of published original data, in large series from 2006-2016, where both surgery and adjuvant therapy were used for the treatment of esthesioneuroblastoma.

**Results:**

Single institution review revealed eight patients with esthesioneuroblastoma, half with advanced Kadish staging. All Kadish A patients ( Kadish A: confined to nasal cavity) underwent endoscopic approaches alone, while Kadish C patients (Kadish C: extends beyond nasal cavity and paranasal sinuses) and D patients (Kadish D: lymph node or distant metastases) underwent craniofacial approaches, while all patients received post-operative adjuvant therapies. Complications such as cerebrospinal fluid (CSF) leak, seizures, meningitis, and abscess only occurred in high Kadish stage patients. Literature review demonstrated a higher proportion of advanced Kadish stage cases correlated with increasing rates of pneumocephalus, infection, and recurrence. A higher proportion of Kadish C and D tumors was inversely correlated with CSF leak rate and overall survival.

**Discussion:**

Advanced stage tumors are often associated with a higher incidence of adverse events up to 33%, both due to disease burden and treatment effect. There is increasing use of endoscopy and neoadjuvant therapy, which have the potential to decrease complication rates.

**Conclusion:**

Advanced Kadish stage esthesioneuroblastoma necessitates meticulous surgical resection and aggressive adjuvant therapies, together, these increase the likelihood of adverse events, including CSF leak, neurologic deficits, and infections, and may represent the real morbidity cost of radically treating these tumors to achieve an improvement in overall survival. In selected patients, less-invasive approaches or neo-adjuvant therapies can be used without compromising on a curative resection.

## Introduction

Esthesioneuroblastoma or olfactory neuroblastoma is a rare, malignant, neuroendocrine tumor arising from olfactory neurosecretory cells near the cribriform plate [1-2]. The first and most widely used staging system was described by Kadish, et al. in 1976 and included three tiers: stage A–disease limited to the nasal cavity, stage B–inclusion of the paranasal sinuses, and stage C–extension beyond the cribriform plate and paranasal sinuses [3]. This was later modified by Morita, et al. and he included stage D–the involvement of cervical lymph nodes and distant metastases [4]. Analysis of large database studies as well as single institution reviews has statistically demonstrated worse overall survival with more extensive modified Kadish staging [5-6].

Multiple studies have demonstrated greater disease-free and overall survival with margin-free resection, such that higher-grade tumors often necessitate more adjuvant and neoadjuvant therapy, as well as larger, more invasive approaches for resection [1-2,7-9]. Intuitively, this can lead to greater rates of complications including cerebrospinal fluid (CSF) leak, pneumocephalus, infections, radionecrosis, and mortality. The rate of complication, both with our own single-institution review of esthesioneuroblastoma patients as well as reported complication rates in the literature were evaluated, as stratified by Kadish staging.

## Materials and methods

### Retrospective review and case presentation

A retrospective chart review was performed for all patients with the diagnosis of esthesioneuroblastoma who were managed medically and/or surgically at the University of Arizona from 2011-2016. Patients with sinonasal undifferentiated carcinomas were excluded from our study. Charts were analyzed for demographic information, the extent of disease, Kadish staging, presenting symptoms, neoadjuvant and adjuvant therapies, surgical approaches, the extent of resection, complications, recurrence, progression-free and overall survival. For our analysis, we grouped the high Kadish score patients together to better compare between studies who use Kadish or modified Kadish-Morita classifications. Due to the small number of patients and retrospective nature of the study, IRB approval was not required by our institution.

###  Literature review

A medical literature analysis and retrieval system online (MEDLINE) search was performed using the search terms: *esthesioneuroblastoma, olfactory neuroblastoma outcome, complication, *and *Kadish*. Studies containing original data from 2006-2016 were selected to represent modern multidisciplinary and multimodal management of the disease. Only studies with original data were included in our review to avoid patient overlap from literature reviews and meta-analyses. For single-institution studies that were published several times, only the most recent publication was evaluated to avoid multiple representations of the same data set. Other exclusion criteria included patients that either did not receive surgical resection or the goal of surgery was not curative, studies that did not report Kadish staging, complications, insufficient data including case reports. Ultimately, we found nine studies that fit our criteria. These studies were extensively reviewed for demographic information, surgical goals, approaches, outcomes, complications, disease extent and modified Kadish staging, the incidence of local cervical lymph node and distant metastatic recurrence, progression-free and overall survival.

## Results

### Retrospective review

A single-institution chart review revealed eight patients diagnosed with esthesioneuroblastoma from 2011-2016. The average age that was presented was 52 years of age (26 years to 72 years) with an equal male to female distribution. Average follow-up was 60.4 months (18-124 months). Most patients presented with nasal congestion, while other symptoms included epistaxis, discharge, sinusitis, anosmia, and headaches. All patients had disease involving the cribriform plate and half involved with the paranasal sinuses. Some patients had a more extensive disease with infiltration into the orbit, intracranial spaces, and even extension into the frontal lobes and infratemporal and parapharyngeal spaces. Half of our patients had disease localized to the nasal cavity (Kadish A) while two patients had each Kadish C and D stage disease as represented in Table 1.

**Table 1:** Patient characteristics at presentation

	Patients (*n=8*)
Demographics:	
Age at presentation (y)	51.8 (26-72)
Male: Female	1:1
Symptoms:	
Nasal congestion	62.5% (5)
Sinusitis	12.5% (1)
Nasal discharge	12.5% (1)
Epistaxis	25% (2)
Anosmia	12.5% (1)
Headaches	12.5% (1)
Disease Extent:	
Cribriform plate	100% (8)
Paranasal sinuses	50% (4)
Intracranial-extradural	25% (2)
Brain invasion	25% (2)
Orbit	12.5% (1)
Infratemporal	12.5% (1)
Parapharyngeal	12.5% (1)
Cervical lymph nodes	25% (2)
Kadish Stage:	
A	50% (4)
B	0 (0%)
C	25% (2)
D	25% (2)

None of the eight patients received neoadjuvant radiation or chemotherapy. All patients with Kadish A disease were treated with the endoscopic approaches, without the addition of transcranial resection, while all patients with Kadish C-D disease were treated with either simultaneous or staged craniofacial approaches, only one of which was not endoscopic-assisted. The choice of approach was made by the surgeons treating. The goal of surgery was curative, margin-free resection in all cases. Histologically negative margins were achieved in six patients, while the other two patients underwent resection at outside institutions and no data regarding the extent of resection was available. All eight patients received post-operative radiotherapy, one initially refused but underwent radiation in a delayed fashion after recurrence and metastatic disease. Two of the patients with Kadish D staging underwent post-operative chemotherapy with platinum-based therapy and etoposide, one in a delayed palliative fashion after the development of systemic metastasis, and the other patient, concurrently with radiation treatment (Table 2). Ultimately, we found an average progression-free interval of 57 months (4-125 months), with overall survival of 88% at the conclusion of our study period, with an average follow-up of 60.4 months (18-124 months).

**Table 2:** Treatment and outcome *One patient initially refused radiation and underwent radiotherapy in a delayed fashion after recurrence **One patient underwent chemotherapy after delayed recurrence with systemic metastases

	Patients (*n=8*)
Surgical approach:	
Endoscopic only	50% (4)
Transcranial only	12.5% (1)
Both	37.5% (3)
Extent of resection:	
Local gross total	75% (6)
Unknown	25% (2)
Adjuvant therapy:	
Radiotherapy	100% (8)*
Chemotherapy	25% (2)**
Recurrence:	
Local	25% (2)
Lymph nodes	12.5% (1)
Distal metastases	25% (2)
Progression-free survival (months)	57.3
Overall survival (months)	87.5
Follow-up (months)	60.4

The complication rate was far greater in those patients with high stage tumors and subsequent transcranial, transbasal level one approaches (Table 3). Complications included stroke, seizures, pneumocephalus, cerebrospinal fluid leak, meningitis, abscess, osteomyelitis, and radionecrosis. The incidence of complications was significantly greater for advanced stage disease (Kadish C-D) than lower stage tumors. All neurologic complications including CSF leak, hydrocephalus, pneumocephalus, stroke, and seizures occurred in our Kadish C and D patients, while no Kadish A patients experienced neurological deficits aside from anosmia. Similarly, infectious complications of meningitis, osteomyelitis, and epidural abscess only occurred in our patients with advanced Kadish stage esthesioneuroblastoma, without any such instances in our Kadish A patients.

**Table 3:** Complications

	Kadish A/B	Kadish C/D
Patients (*n*)	4 (50%)	4 (50%)
Surgical approach		
Endoscopic only	4 (100%)	0 (0%)
Transcranial only	0 (0%)	1 (25%)
Both	0 (0%)	3 (75%)
Complications:		
1. Neurologic		
CSF Leak	0 (0%)	2 (50%)
Hydrocephalus	0 (0%)	2 (50%)
Pneumocephalus	0 (0%)	1 (25%)
Stroke	0 (0%)	1 (25%)
Seizure	0 (0%)	2 (50%)
2. Radiation		
Cataracts	1 (25%)	0 (0%)
Dermatitis	1 (25%)	0 (0%)
Radiation Necrosis	1 (25%)	0 (0%)
3. Infectious		
Meningitis	0 (0%)	1 (25%)
Epidural Abscess	0 (0%)	1 (25%)
Osteomyelitis	0 (0%)	3 (75%)
4. Recurrence		
Local	1 (25%)	1 (25%)
Cervical Lymph Node	0 (0%)	1 (25%)
Distant Metastases	1 (25%)	1 (25%)
5. Death	0 (0%)	1 (25%)

### Literature review

**Figure 1:**
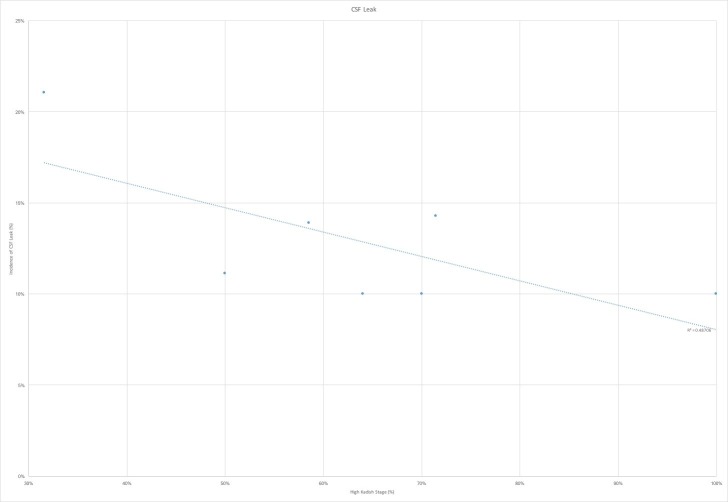
Cerebrospinal fluid CSF leak by high Kadish stage proportion CSF leak stratified by proportion of high-Kadish stage (%) shows an inverse correlation between proportion of high Kadish stage tumors and rates of CSF leak. R^2 ^represents the correlation coefficient of the linear regression

**Figure 2:**
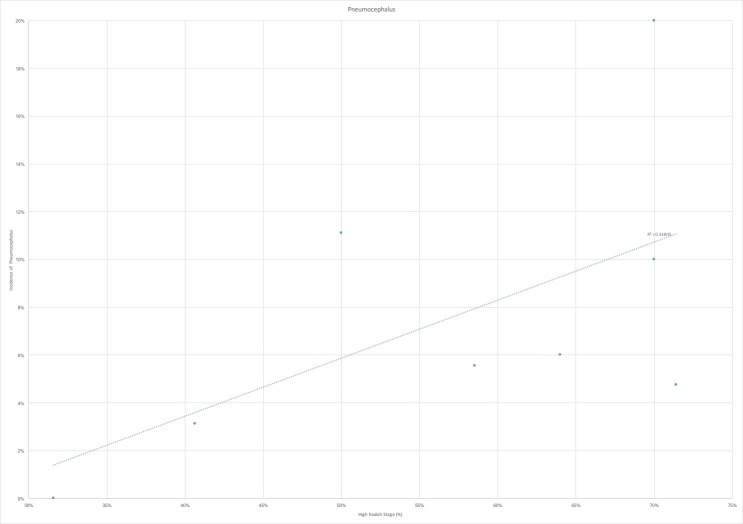
Pneumocephalus by high Kadish stage proportion Pneumocephalus stratified by proportion of high-Kadish stage (%) shows a positive correlation between proportion of high Kadish stage tumors and rates of pneumocephalus. R^2 ^represents the correlation coefficient of the linear regression

**Figure 3:**
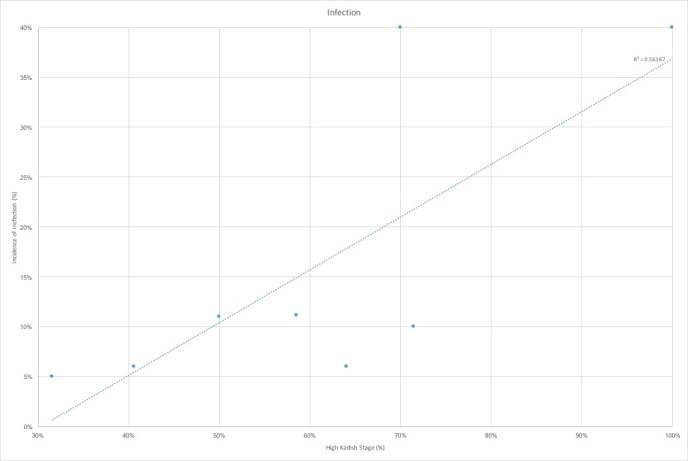
Infections by high Kadish stage proportion Infections stratified by proportion of high-Kadish stage (%) shows a positive correlation between proportion of high Kadish stage tumors and rates of infection, including meningitis, osteomyelitis and epidural abscess. R^2 ^represents the correlation coefficient of the linear regression

The nine studies that were ultimately selected were analyzed for incidence of the cerebrospinal fluid leak, pneumocephalus, meningitis, abscess formation, osteomyelitis, local recurrence, cervical lymph node recurrence, distant metastases and overall survival [1,7,9-13]. In the seven studies that had reported CSF leak rate, we found a trend towards lower incidence of the leak in those studies with a larger proportion of highest Kadish tumors (Figure 1). This can potentially be attributed to the use of open craniofacial and cranionasal approaches in higher stage tumors, which can allow for broader access for reconstruction. This is, however, in contradistinction to our study, where the CSF leak rate was 200% greater in cranionasal approaches than purely endoscopic resections. We also found a trend towards increased rates of pneumocephalus with the higher proportion of Kadish C and D tumors (Figure 2). This is likely attributable, again, to the greater proportion of open approaches for advanced stage tumors. Across all infectious outcomes evaluated, the eight case analysis showed correlation for higher rates of infection, including meningitis, osteomyelitis, and abscess with a greater proportion of Kadish C and D tumors (Figure 3).

**Figure 4:**
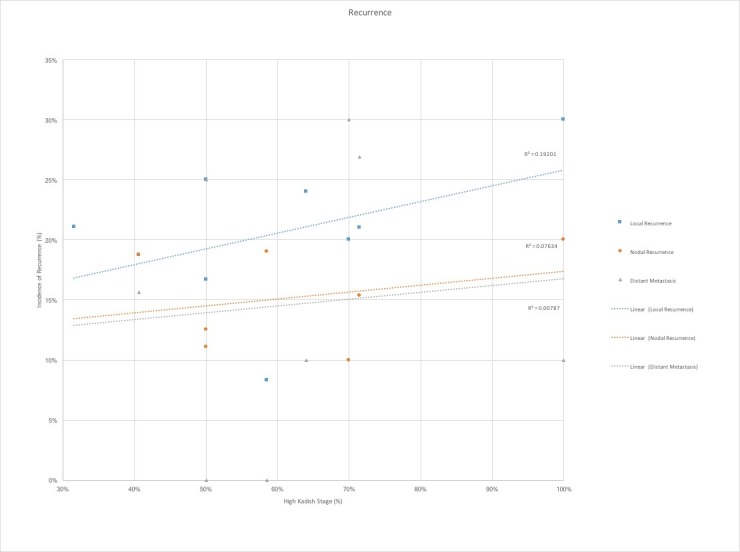
Recurrence by high Kadish stage proportion Local, cervical lymph node, and distal metastatic recurrence stratified by proportion of high-Kadish stage (%) shows a positive correlation, greatest for local recurrence and only minimally positive for lymph node and distal metastatic recurrence. R^2 ^represents the correlation coefficient of the linear regression

**Figure 5:**
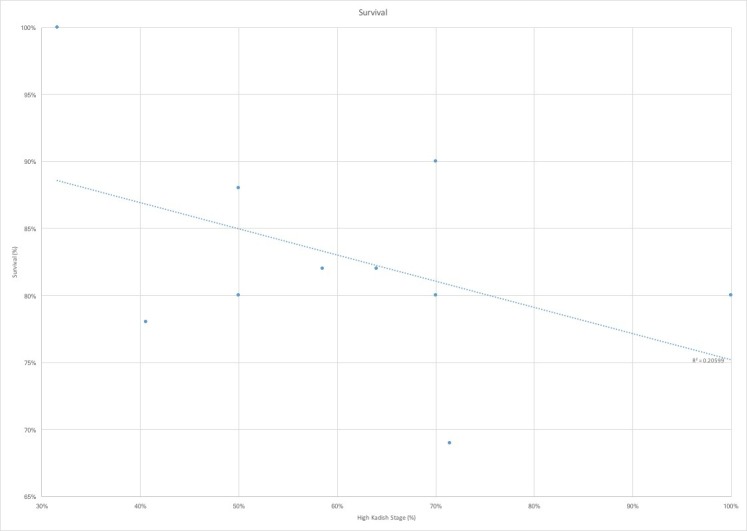
Overall survival by high Kadish stage proportion Survival stratified by proportion of high-Kadish stage (%) shows an expected inverse correlation between proportion of high Kadish stage tumors and overall survival. R^2 ^represents the correlation coefficient of the linear regression

We observed a trend towards increased rates of recurrence, including local, cervical lymph node, and distal metastatic recurrence, with the greater proportion of higher stage tumors (Figure 4). Finally, the greatest percentage of Kadish C and D stage tumors correlated with decreased survival rates (Figure 5). The average follow-up of the studies evaluated was only 60 months, which grossly underestimates rates of recurrence and mortality, as esthesioneuroblastoma has been proven to have significantly delayed recurrences [1].

## Discussion

Most cases of esthesioneuroblastoma are diagnosed at later stages, at which point the tumor is already quite extensive, largely due to its nonspecific symptoms and overall rarity, which keep clinicians’ suspicions low [3,14-15]. Since most esthesioneuroblastomas have the greater extension and even intracranial invasion at the time of diagnosis, these advanced stage tumors become challenging to treat [1-2,4-5,14,16]. The Kadish staging system and its later modification considers radiographic tumor extent and provides important clinical information that can guide clinicians with the surgical approach, types of adjuvant therapy and prognosis [3-4]. High Kadish classification, particularly with cervical node metastases has been shown to be a strong negative prognosticator [4,6]. Cervical lymph node metastases have been reported to range from 17%-33% (average being 23%) [17-18]. The presence of cervical disease is a strong poor prognosticator with 29% overall survival in those with nodal disease and 64% in those without. The rate of distal metastatic disease was found to raise from 51% to 86% in the presence of cervical lymph nodes [19].

Radical surgical resection with disease-free margin is a strong predictor of tumor freedom and prevention of significant delay of both local and distal recurrence [8,10,14,20]. Positive surgical margins in anterior skull base tumors were found to double local recurrence rates and reduce the survival as half [10,20]. The use of craniofacial approaches led to over a two-fold improvement in disease-free survival [6,10,20-21]. As expected, after its introduction, craniofacial resection became the gold standard in the treatment of esthesioneuroblastoma [2,6–8,10,13]. Craniofacial resection increased progression-free-survival from 37.5% to 82% in comparison to extracranial resection [6,10,20-21]. However, post-operative complication rates for craniofacial approaches remain high. Results from an international collaborative study show complication rates of 33% for anterior craniofacial resection, where the wound complications were the most common and are present in 18% of patients [20]. Higher risk of adverse events was associated with prior radiation treatment and greater intracranial tumor extent [20,22]. Our study reflected these findings as those with more advanced Kadish C and D stage disease that had significantly worse outcomes including seizures, strokes, osteomyelitis, and abscesses that require multiple revision surgeries in some cases. Review of the literature also reflected a correlation between the incidence of pneumocephalus and infection with higher proportions of advanced Kadish stage tumors.

There are several tactics that can be taken to reduce the overall morbidity associated with advanced stage tumors. Many have shown that minimizing the surgical footprint with more minimal approaches without compromising extent of resection could benefit major complication rates [1-2].

Yuen, et al. first described the use of an endoscopic approach in the treatment of esthesioneuroblastoma in 1997 [23]. Since then, multiple studies have not only reproduced its feasibility but, in some cases, demonstrated its superiority [2,7-8,10]. The use of endoscopy can provide greater visualization of tumor margins and vital structures, as well as preserve cosmesis [24]. Equipoise and in some cases, superiority has been demonstrated with endoscopic-only or endoscopic-assisted approaches, as compared to transcranial approaches for complete margin-free resection, albeit with the ever-present caveat of careful patient selection [25].

Endoscopic approaches can either be classified as purely endoscopic or endoscopic-assisted craniofacial resections. Several series, including our own, show excellent results with endoscopic-assisted approaches, including clear surgical margins and long-term disease-free and overall survival with minimal adverse events [2,7-8,10,13]. Many studies report significantly lower rates of complications in those patients undergoing endoscopic-only surgical approaches, in contrast, to open transcranial or trans facial surgical resection [2,7-8,12]. In some cases, endoscopic-only approaches did not compromise the extent of resection in even Kadish C disease [7], though many studies did not produce the same results [8,10,12]. This finding was comparable to our results, where our patients undergoing endoscopic-only approaches had fewer complications. The obvious reasoning for this is that disease extent begets the approach and the goal of treatment should always be complete resection [8]. In our institution, those with limited disease, specifically Kadish A, where the treating surgeons felt complete resection was feasible from purely endoscopic approaches, were treated as such. Those with the worse disease had more extensive surgical approaches and higher rates of complications ensued. This can be attributed to both their disease burden, more extensive surgery and the need for adjuvant therapies.

One major concern for the endoscopic-only approach is the incidence of CSF leak. Our review of the literature indicated that CSF leak incidence was greater with lower Kadish stage disease. One hypothesis for this is the less advanced disease is more likely to be treated with endoscopic only approaches in the modern era, leading to potentially greater leak rate. However, with advancements in multilayer endonasal skull base reconstructions, this has become of a less concern [2,7]. In fact, our own study indicated there was a higher incidence of fluid leak from combined endoscopic and craniofacial approaches. This is likely attributable to more extensive resections required by the advanced disease stages and the use of postoperative radiation and chemotherapy that decreases the integrity of the reconstruction, but is nevertheless necessary for disease control and survival.

Single-modality treatment, i.e. radiation or surgery in isolation was found to have worse outcomes and higher rates of local recurrence and distal metastases [14-15]. Dulguerov, et al. performed a meta-analysis of 26 studies including 390 patients which ultimately showed the best outcomes in esthesioneuroblastoma were when surgery was followed by radiation treatment [26]. Several large studies have also shown optimal treatment to be radical surgical resection and radiation therapy [1]. Some groups argue for aggressive therapy with surgical resection, radiation, and chemotherapy to achieve the highest rates of disease freedom [14]. Multimodality treatment is particularly advocated for high Kadish stage disease [15].

The addition of chemotherapy, however, has been controversial, where many groups, including our own initial experience, reserve its use for recurrent or systemically disseminated disease [10]. However, most recent studies have shown a benefit for patients with high Kadish stage disease and our own practices have shifted to perform concomitant chemotherapy in the postoperative period for these advanced cases [27]. Most chemotherapy regimens include the use of etoposide and a platinum-based agent such as Cisplatin or Carboplatin [15]. Other regimens include the use of cyclophosphamide and vincristine with good long-term control rates [1,21,28]. Both have shown good results.

Another area of concern for advanced Kadish stage esthesioneuroblastomas has been the presence of lymph node involvement. The rates of cervical lymph node metastases have been reported as high as 33% and can present nearly a decade, after the control of the primary disease [17-18]. Advanced Kadish stage disease, even with adequate local control has been shown to have delayed cervical node metastases in up to 16% of cases, only one-third of who are salvaged with additional therapy [17]. Because of this, some argue in favor of and have shown good results with prophylactic neck irradiation in locally aggressive Kadish B and C disease [18]. Others reserve the use of cervical radiation, used in conjunction with neck dissection, for cases of disease recurrence [1]. Our study did not involve neck dissection for the two patients with Kadish D disease, one due to surgical feasibility and other due to his palliative status. However, we offered cervical radiation for both patients, though none of the patients had prophylactic irradiation.

The goals of oncologic treatment, especially in the modern era, are to improve rates of curative treatment and disease freedom while minimizing treatment impact and associated complications. This is especially challenging with aggressive malignant tumors such as esthesioneuroblastomas, that are both locally and distally aggressive with the potential of long-delayed recurrences. One method of decreasing tumor burden and resultant surgical extent of resection is the use of neoadjuvant therapy. The University of Virginia team, in particular, advocates the use of neoadjuvant therapy [1,21,28-29]. Their most recently published series includes 50 patients who had strict adherence to their protocol of neoadjuvant therapy. All Kadish A and B patients underwent neoadjuvant radiotherapy, while Kadish C patients had pre-operative radiation and chemotherapy. They reported an 87% and 83% of five and 15-year disease-free survival, respectively, which is significantly higher than studies that show the “gold standard” approach of complete craniofacial resection followed by adjuvant radiotherapy [1,26]. One of the drawbacks of neoadjuvant therapy is the confounding nature of radiation changes on histopathology which can impede obtaining clear surgical plans and histological margin-free resections [16].

As noted by most studies, the ability to perform large-scale analyses for esthesioneuroblastoma is challenging due to the relative scarcity of the tumor type [2,25-26]. Several studies have also evaluated the surveillance, epidemiology, and end results (SEER) database in an effort to achieve larger conclusions with greater statistical power [5,30]. However, most of our information comes from multiple single institution reviews that use different grading scales, surgical approaches and goals, and treatment algorithms for both adjuvant and neoadjuvant therapies [1,6,10-11,21,28-29]. Similarly, one of the limitations of our study is the fairly small sample size of only eight patients and relatively short follow-up (median 60.4 months, range 18-124 months). Additionally, our literature review included very few studies as most studies did not clearly report complication rates, use Kadish staging, or strive for margin-free resections. We also narrowed the scope of our literature search to include only those studies that would utilize modern techniques including endoscopic approaches (and leak repair), intensity-modulated radiation therapy (IMRT), gamma knife stereotactic radiosurgery, neoadjuvant therapies, etc. Nevertheless, our study does stress the higher incidence and prevalent types of complications that can be encountered with advanced Kadish stage esthesioneuroblastoma and should be considered when selecting the appropriate treatment regimens for such patients.

## Conclusions

The goals of esthesioneuroblastoma should always include curative, clear-margin resection. This is obviously more complicated in more advanced, higher Kadish stage tumors. More of extensive disease, meticulous surgical resection, and aggressive adjuvant therapies together increase the likelihood of adverse events, including CSF leak, neurologic deficits, and infectious complications. Some tactics to potentially decrease the morbidity of high stage tumors and their treatment includes neoadjuvant therapy and minimally invasive approaches without compromising extent of resection. The gold-standard in the treatment of esthesioneuroblastomas remains as an extensive resection with a goal of surgical cure, addition of adjuvant therapies and the management of ensuing complications when they arise.
